# Glycated haemoglobin versus fasting plasma glucose for type 2 diabetes point of care screening: a decision model cost-effectiveness analysis

**DOI:** 10.1186/s12913-025-12840-4

**Published:** 2025-05-09

**Authors:** Francis Xavier Kasujja, Meena Daivadanam, Roy William Mayega, Fred Nuwaha, Ronald Kusolo, Elizabeth Ekirapa

**Affiliations:** 1 Department of Epidemiology and Biostatistics, Makerere University, P. O. Box 7072, New Mulago Hill Road, Mulago, Kampala, Uganda; 2 Chronic Diseases and Cancer Theme, MRC/UVRI & LSHTM Uganda Research Unit, Entebbe, Uganda; 3 Global Health and Migration Unit, Department of Women’s and Children’s Health, International Maternal and Child Health, Uppsala University, Uppsala, Sweden; 4 Department of Global Public Health, Karolinska Institutet, Solna, Sweden; 5 Department of Disease Control and Environmental Health, Makerere University, P. O. Box 7072, New Mulago Hill Road, Mulago, Kampala, Uganda; 6 Department of Health Policy Planning and Management, Makerere University, P. O. Box 7072, New Mulago Hill Road, Mulago, Kampala, Uganda

**Keywords:** Cost-effectiveness analysis, Diabetes tests, HBA1c, FPG, Diabetes detection

## Abstract

**Introduction:**

Whereas fasting plasma glucose (FPG) is cheaper, the glycated haemoglobin (HBA1c) test, which does not require fasting, is more convenient for diabetes screening and could be available to patients throughout the day. In this study, we compared the cost effectiveness of the HBA1c test to that of the FPG test when used for point-of-care (POC) screening of type 2 diabetes in a low-resource setting in Uganda.

**Methods:**

A cost-effectiveness analysis from a societal perspective was conducted for a single screening cycle of 1659 adults aged 35–70 years receiving care at the outpatient department of a general hospital. We constructed a decision analysis model using TreeAge Pro Healthcare v2023, with the cost estimated using an ingredient approach and the effectiveness measured based on the proportion of patients correctly diagnosed with diabetes.

**Results:**

The unit cost was US$ 6.48 for the HBA1c test and US$ 8.39 for the FPG test. However, a marginally greater percentage of patients were correctly diagnosed according to the FPG test (96.3%) than the HBA1c test (96.2%). The cost-effectiveness ratio was $6.74 for the HBA1c test and $8.39 for the FPG test. The incremental cost effectiveness ratio was $989.06 per additional patient correctly diagnosed with diabetes.

**Conclusion:**

HBA1c POC testing could be a more cost-effective alternative to the FPG POC test for the screening of diabetes in under-served outpatient populations in Uganda and similar contexts.

**Supplementary Information:**

The online version contains supplementary material available at 10.1186/s12913-025-12840-4.

## Introduction

Type 2 diabetes is one of the most prevalent noncommunicable diseases (NCDs) worldwide. Low- and middle-income countries, where 3 in 4 diabetes patients live, face the brunt of the epidemic, with the disease incidence projected to increase by 134% to 55 million people by 2045 [1]. The absolute and relative mortality rates associated with diabetes in sub-Saharan Africa (SSA) are highest in the 20–39 year age group [2], which is the most economically productive. However, more than half of all individuals with diabetes in SSA are unaware of their glycaemic status [3] which predisposes them to diabetes complications and premature mortality.

Improving diabetes detection in under resourced settings requires the at-scale deployment of early diagnosis interventions centred on cost-effective diabetes testing. The glycated haemoglobin (HBA1c) and fasting plasma glucose (FPG) tests are two of the most common diabetes tests. The World Health Organization (WHO) recommends the use of point-of-care (POC) FPG and HBA1c analysers for diabetes screening and diagnosis in settings where central laboratory facilities are inadequate or unavailable [4, 5].

The FPG test, which forms the diabetes testing standard in public health facilities in Uganda and similar settings, requires individuals to fast for 8–14 h. Its use is often restricted to morning hours to ensure that patients are fasted before admission. The HBA1c test, on the other hand, does not require fasting. It is therefore available to patients throughout the day without the need for them to return to the health facility in a fasted state. Other advantages of HBA1c include its ability to provide a measure of the average glycaemic level over the previous 2–3 months as well as its greater reproducibility [6]. However, most countries in SSA have yet to embrace HBA1c testing, citing concerns about the lack of evidence regarding test performance in the local setting, as well as its cost.

Emerging evidence from studies investigating SSA among various clinical subpopulations points to HBA1c performing comparably to the FPG test [7–11]. This finding points to a potential role for the HBA1c test, either alone or alongside the FPG test, in diabetes screening and diagnosis. Nonetheless, questions regarding the cost effectiveness of the HBA1c test compared to the FPG test linger. This is unsurprising considering that up to 20% of the monthly public health service costs for diabetes care have been linked to medical tests [12], yet HBA1c testing is generally more costly than FPG testing is.

However, research conducted elsewhere suggests that HBA1c testing can be more cost-effective than FPG testing [13]. Therefore, judicious assessment of the costs and health benefits of HBA1c and FPG testing is warranted to understand which of the two tests would be preferable for diabetes testing in Uganda and similar contexts. This study was conducted to compare the cost effectiveness of HBA1c testing to that of FPG testing when used for point-of-care testing of type 2 diabetes in a low-resource setting in eastern Uganda.

## Methods

### Study setting

The study was undertaken in the outpatient department of Iganga Hospital, a general hospital in eastern Uganda. The hospital is located 120 km from Kampala along Jinja-Tororo Road, which is a busy transport corridor linking northeastern Uganda and Kenya to Kampala, the capital city of Uganda. It serves communities in Iganga district and the contiguous parts of Luuka, Mayuge, Bugweri, Bugiri, Namutumba and Kaliro districts. Most of these communities are rural and depend on subsistence farming for their livelihood. Less than 20% of the households live within 5 km of the nearest public health facility [14].

In addition to inpatient services, the hospital operates a busy outpatient department (OPD) for patients referred from lower-level health facilities. However, most patients seen at the hospital are self-referrals. The outpatient department provides general curative and maternity services. Whereas FPG testing is available at the hospital’s diabetes clinic, HBA1c testing facilities are not available. Diabetes screening services are not available at the hospital. Because of this, diagnosis often occurs after patients have developed overt diabetes symptoms [15, 16].

### Study design

This was an economic evaluation comparing HBA1c and FPG testing, which was nested in a diagnostic accuracy study. The analysis was based on the societal perspective, with the analytic horizon starting at the point of screening of an eligible outpatient and ending at the time when a diagnostic decision was made.

### Participant selection, sampling strategy, and sample size

The methods used for participant selection and the sampling strategy used for the sample used for the measurement of the effectiveness, as well as the direct costs, are described in detail elsewhere [9]. Briefly, patients eligible for inclusion were adults aged 30–75 years who were receiving care at the outpatient department of a general hospital in eastern Uganda. Individuals known to have diabetes and who were receiving antihyperglycemic drugs, antipsychotic drugs, or systemic steroids were excluded. The same was done for those known to have sickle cell disease or with clinical features suggestive of sickle cell disease, as well as those with a history of having undergone blood transfusion within the previous 3 months.

A two-stage sampling strategy was used, whereby consecutive sampling and FPG testing were initially conducted for all participants. This was followed by test result-based sampling. The latter involved subsequent testing using the HBA1c and OGT tests for two categories of patients: every participant who scored more than 6.0 mmol/l on the FPG test and the next individual with a score of 6.0 mmol/l or less to ensure a 1:1 ratio. In total, 1659 individuals were sampled, and tested using FPG; 310 of these individuals subsequently underwent HBA1c and oral glucose tolerance (OGT) testing.

### Description of testing alternatives

FPG POC testing was the comparator, and HBA1c POC testing was the intervention. FPG testing was conducted using a handheld Accu-Chek^®^ Active blood glucose meter (Roche Diagnostics GmbH, Mannheim, Germany). HBA1c testing using a Cobas b101 benchtop HBA1c analyser (Roche Diagnostics GmbH, Mannheim, Germany). The two tests were gauged against the OGT test. The latter was measured using an Accu-Chek^®^ Active glucometer from a capillary blood sample taken 2 h after patients had fasted; 75 g of anhydrous glucose was dissolved in 250 ml of water.

Both the Accu-Chek^®^ Active glucometer and the Cobas b101 analyser meet international standards. The Accu-Chek^®^ Active glucometer is calibrated to measure plasma glucose levels from whole blood specimens. It meets ISO 15197:2013 specifications. At least 95% of the glucometer results are within ± 15 mg/dl at glucose concentrations < 100 mg/dl and within ± 15% at ≥ 100 mg/dl compared to a traceable laboratory method [17]. The Cobas b101 HBA1c analyser, on the other hand, is accredited by the National Glycohemoglobin Standardization Program (NGSP) and standardized to the Diabetes Control and Complications Trial (DCCT) assay. The coefficient of variation of the Cobas b 101 instrument is less than 5% [18], in line with the recommendations of the International Federation of Clinical Chemistry and Laboratory Medicine for HBA1c POC tests [19, 20]. It is also stable to interference from sickle cell haemoglobin variants.

### Measurement of effectiveness

The sensitivity, specificity, and prevalence of diabetes were derived from a previous study [9] using the test cut-off points for type 2 diabetes recommended by WHO [21] and the American Diabetes Association (ADA) [22] for diabetes. For either test alternative, four outcomes were possible: true positive, true negative, false positive and false negative. True-positive and true-negative blood samples were those that tested positive or negative on either the HBA1c or FPG tests or the OGT test, respectively. On the other hand, false-positive and false-negative blood samples tested positive or negative on either test alternative but not on the OGT test.

### Measurement of cost

The costing process involved the collection of study cost data, interviewing study staff, health facility staff (Additional file 5), and the review of public records. The data included the internal costs that would be incurred by the Ministry of Health. All estimates were performed based on the costs on June 1, 2019, and were originally estimated in Uganda Shillings (UGX) and subsequently converted to United States dollars (1 USD = 3789.58 UGX on June 1, 2019) adjusted to the year 2023 [23].

For the two test alternatives, the ingredient (bottom-up) approach was used to determine the costs that would be incurred if the same patient were tested using either FPG or HBA1c. This involved the observation of testing procedures and reading of standard operating procedures to quantify and value all the inputs. This microcosting was conducted to identify all the test inputs and specific quantities required to perform a single test for both the FPG and HBA1c while excluding the cost of the OGT test, which was only used as a clinical reference standard.

Both capital costs and recurrent costs were considered for this analysis. The capital costs were those considered to be those spent on items with more than one year of useful life. These included diagnostic equipment, furniture, and space. For the diagnostic equipment, the costs of the Cobas b 101^®^ HBA1c analyser (Roche Diagnostics GmbH, Mannheim, Germany) and the Accu-Chek Active^®^ glucometers (Roche Diagnostics GmbH, Mannheim, Germany) were considered. These products were obtained from Medisell Uganda Limited, the sole distributor of Roche products in Uganda. Annualized values of the HBA1c analyser and glucometers were estimated using a standard procedure [24], assuming a useful life span of 5 years [25]. The costs and health benefits were not discounted because the time horizon was a single round of testing, lasting an average of 6 min for the HBA1c test and 2 min for the FPG test. The costs of the furniture and the space were excluded, as their costs were similar for both tests.

The recurrent costs included the cost of test kits, gloves, cotton, and other sundries, as well as labour and utility costs. The Cobas b 101 HBA1c test kits (Roche Diagnostics GmbH, Mannheim, Germany) were obtained from Medisell Uganda Limited. The power consumption was estimated based on the specifications of the HBA1c analyser and costed according to the tariff structure of Umeme Limited, Uganda’s main power distribution company. The costs of lancets, gloves, and alcohol swabs were added to the cost of the test kits to determine the cost of either testing procedure. The costs of the health management information system, laboratory registers, and stock cards were excluded for this analysis.

Estimates for personnel costs per single test were computed based on the time required to conduct a single test. FPG testing was conducted by a nurse, and HBA1c testing was conducted by a laboratory technician. We found that whereas it took 2 min to conduct a single FPG test, the corresponding duration for conducting an HBA1c test was 6 min. The salaries were estimated from the Public Service Salary Structure for 2020 [26], assuming that staff work for 260 days annually, after excluding public holidays, statutory rest days, and leave days.

Patient costs were derived from Shiri et al. [12]. The Shiri study was an economic evaluation of costs for the delivery of outpatient integrated services for diabetes, hypertension, and HIV. The costs from this study were considered a close approximation of the costs for transport, lost productivity and meals for patients receiving care within the outpatient department. We accounted for one trip to the health facility for patients tested using the HBA1c test. For patients tested using the FPG test, we assumed an extra trip, as most patients had eaten some food within the previous 8 h of their initial contact with the health provider. This means that they would have to return to the health facility on another day to undergo testing before their first meal of the day.

### Decision analysis model for cost-effectiveness analysis

**Fig. 1:**
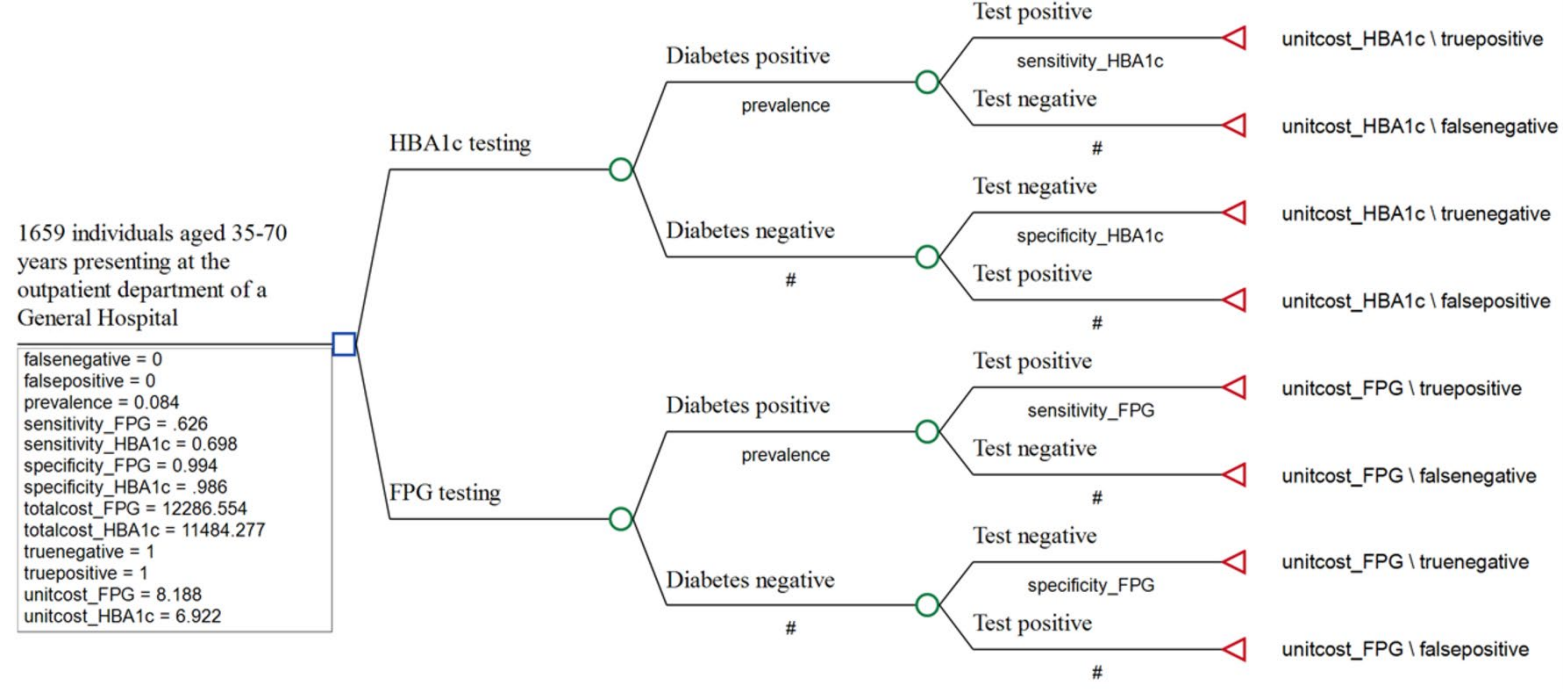
Decision analysis model for the cost-effectiveness analysis of the HBA1c and FPG tests. Legend: Detection pathway: OGT POC test used to determine accuracy of diabetes negative and positive results as diagnosed by FPG and HBA1c POC tests

Cost effectiveness analysis was conducted using a decision tree model (Fig. 1), which was constructed using TreeAge Pro Healthcare 2023 (TreeAge Pro, Inc., Williamston, Massachusetts, USA), to compare the diagnostic outcomes and costs of the HBA1c and FPG tests. The decision tree model is presented in Fig. 1. Individuals with diabetes were tested as either true positives or false negatives, with the corresponding proportion depending on the sensitivity of the test. Individuals without diabetes tested either as true negatives or as false positives, depending on the specificity of the test.

Effectiveness probabilities and values, together with the societal costs incurred for both tests, were used to populate the model to determine its cost effectiveness. The payoffs for the correctly diagnosed cases were based on the proportion of correctly diagnosed individuals for each staining technique. Cases that were correctly diagnosed (true positive and true negative cases) were assigned a payoff of 1, whereas incorrectly diagnosed cases (false positive and false negatives) were assigned a payoff of zero. In both cases, the cost payoffs were the unit costs for each test.

### Cost-effectiveness outcome measure

The cost-effectiveness outcome measure was the incremental cost per additional diabetes test diagnosed, expressed as the incremental cost effectiveness ratio (ICER). The ICER was defined as the change in costs over the change in the effectiveness of moving from FPG to HBA1c expressed as USD per diabetes patient diagnosed. The cost per diagnostic outcome was used to obtain a proximal measure for exploring the key drivers of cost-effectiveness.

### Sensitivity analysis

Sensitivity analysis was also conducted to determine the robustness of the model to changes in the diagnostic accuracy of the HBA1c and FPG tests and to assess the effect of uncertainties in the model parameters on the results. One-way deterministic sensitivity analysis and threshold analysis were also conducted for this purpose. This involved the assignment of low- and high-range limits to the prevalence, sensitivities, and specificities of the two tests based on the upper and lower bounds of the corresponding 95% confidence intervals. The effect on the ICER of running multiple HBA1c analyzers and FPG glucometers in parallel, as well as increasing the number of personnel conducting testing, was also assessed.

## Results

### Cost per test for the FPG and HBA1c tests

The details of the cost of HBA1c and FPG testing are provided in the Additional files (Additional Tables 1-7 in Additional file 1 and Additional Tables 8-11 in Additional file 2).

The FPG test had a greater overall cost ($12,286.55) than the HBA1c test ($11,484.28). Patient costs contributed $9,970.76 (90.4%) to the total cost of FPG testing, whereas they contributed $7,453.89 (64.9%) to the total cost of HBA1c testing (Table 1).

**Table 1:** The costs of FPG and HBA1c testing

	Item	FPG test			HBA1c test			Source
	Cost item	Total cost, US$	Unit cost, US$	%	Total cost, US$	Unit cost, US$	%	
Provider costs	Personnel	5.201	0.003	0.0%	15.332	0.009	0.1%	Primary data
	Equipment	3.790	0.002	0.0%	184.870	0.112	1.6%	
	Power	2.508	0.002	0.0%	0.780	0.000	0.0%	
	Test kits	972.234	0.586	7.2%	3,516.786	2.120	30.6%	
	Consumables	312.622	0.188	2.3%	312.622	0.188	2.7%	
Subtotal, provider costs (US$)		1296.355	0.781	9.5%	4030.390	2.429	35.1%	
Patient costs	Transport costs	4422.894	2.666	32.6%	2211.447	1.333	19.3%	Shiri et al. [12]
	Lost productivity	2573.109	1.551	18.9%	1715.406	1.034	14.9%	
	Meals	5290.551	3.189	38.9%	3527.034	2.126	30.7%	
Subtotal, patient costs (US$)		9970.756	7.407	90.4%	7453.887	4.493	64.9%	
Total unit cost for a societal perspective (US$)		12286.554	8.188	100.0%	11484.277	6.922	100.0%	

### Effectiveness and cost-effectiveness

The parameters for the decision model are summarized in Table 2.

**Table 2:** Decision model parameters

Parameter	Base case value	Low	High
Prevalence of diabetes*	8.4%	7.09%	9.82%
True positive payoff	1		
True negative payoff	1		
False positive payoff	0		
False negative payoff	0		
HBA1c test			
Sensitivity*	69.8%	46.3%	86.1%
Specificity*	98.6%	95.4%	99.6%
Unit cost of HBA1c testing	US$ 6.922		
FPG test			
Sensitivity*	62.6%	41.5%	79.8%
Specificity*	99.4%	98.9%	99.7%
Unit cost of FPG testing	US$ 8.188		

*Source: Kasujja et al. [9]

**Fig. 2:**
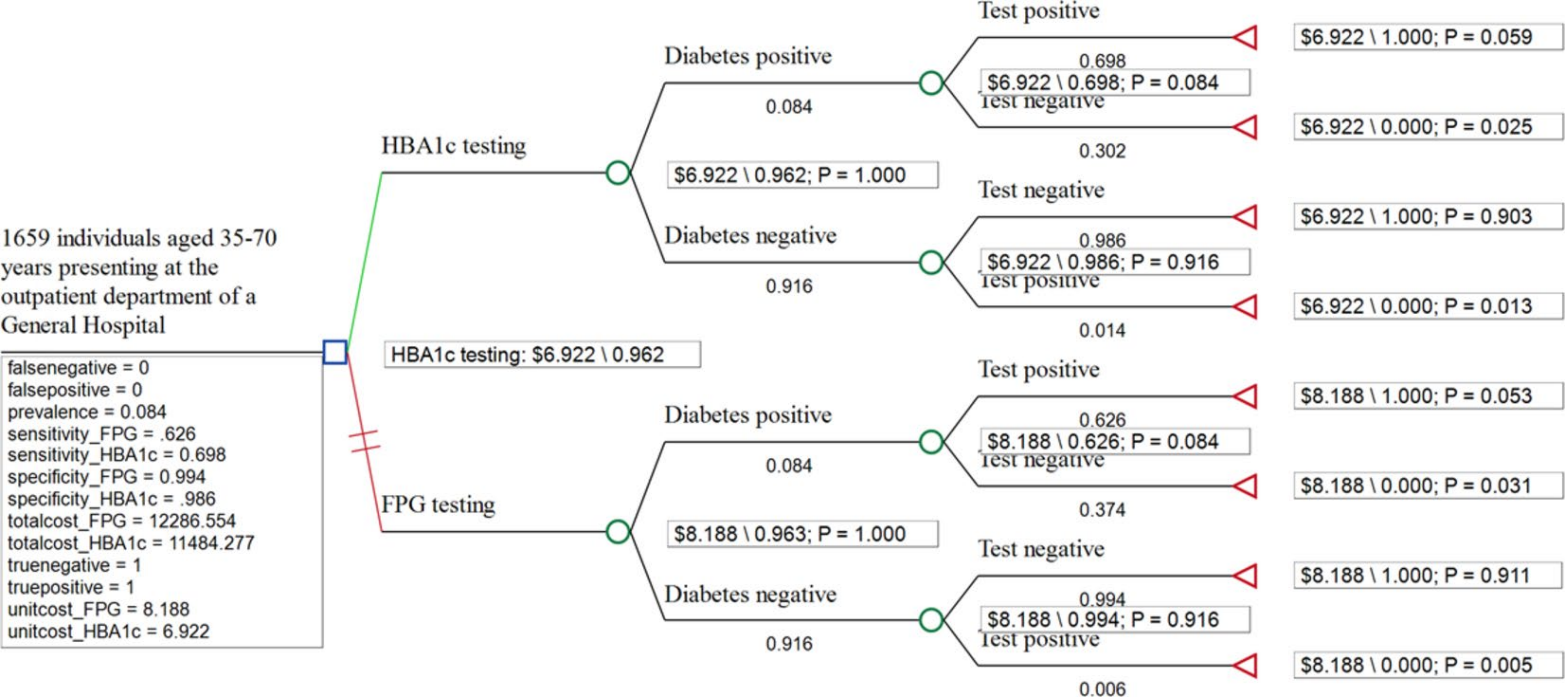
Rolled-back decision analysis tree comparing HBA1c and FPG

The proportions of patients correctly diagnosed by both the FPG (96.3%) and HBA1c (96.2%) tests were comparable (Table 3 and Fig. 2).

**Table 3:** Cost effectiveness rankings

Tests	Costs (US$)	Effectiveness	Incremental costs (US$)	Incremental effectiveness	ICER (US$)
HBA1c	6.48	0.962			
FPG	8.39	0.963	-1.91	-0.001	989.06

The ICER was $989.06 per additional individual correctly diagnosed (Table 3).

### Sensitivity analysis

According to the one-way sensitivity analysis, the variable with the highest impact on the ICER was prevalence (Additional Fig. 1 in Additional file 3), with the ICER ranging from $774.15 to $5,616.92. However, the ICER was robust to values within the uncertainty range of prevalence (Table 4). The proportion of individuals correctly diagnosed with diabetes was equal for individuals screened with the HBA1c and FPG tests if the HBA1c sensitivity, HBA1c specificity, FPG sensitivity or FPG specificity were 71.3%, 98.7%, 61.1% or 99.3%, respectively.

**Table 4:** One-way sensitivity analysis/threshold analysis

Variable	Baseline (%)	Range (%)	Threshold value (%)*	Sensitive? **
Prevalence of diabetes	8.4	7.09–9.82	NA	No
HBA1c sensitivity	69.8	46.3–86.1	71.3	Yes
HBA1c specificity	98.6	95.4–99.6	98.7	Yes
FPG sensitivity	62.6%	41.5–79.8	61.1	Yes
FPG specificity	99.4%	98.9–99.7	99.3	Yes

*Parameter value (within uncertainty range) at which both comparators are equal

**A strategy other than the optimal one (in base case analysis) is preferred at some point in the range, i.e., the threshold value is within the range. Otherwise, the result is not sensitive, i.e., it is robust

The ICER decreased with increasing values of FPG sensitivity and specificity within its uncertainty range. Conversely, the ICER increased with increasing diabetes prevalence, HBA1c specificity and HBA1c sensitivity (Additional file 3, Fig. 2). Running tests on multiple instruments would lower the ICER, with two parallel instruments resulting in an ICER of $903.15 compared to $817.19 for 3 parallel instruments. The ICER would also be further reduced if more healthcare workers participated in diabetes screening. For example, having 2 healthcare workers running 4 testing devices would result in an ICER of $726.56.

Sensitivity analysis further showed that a cheaper HBA1c analyser would reduce the provider costs and overall cost of HBA1c testing. This would result in a higher incremental cost. Assuming the diagnostic accuracy of HBA1c on the new device were comparable to that of the Cobas b101 analyser, the incremental effectiveness between FPG and HBA1c testing would not be affected, resulting in higher values of the ICER. For example, if the overall provider cost of HBA1c testing were equal to the current provider cost of FPG testing ($0.781), the ICER would be $2,276.56, assuming no changes in patient costs for either test. In this case, adoption of HBA1c testing for diabetes screening would be guided by whether the WTP threshold.

However, if the assay of the new HBA1c analyser had better diagnostic accuracy than the Cobas b101 device and its provider costs were similar to those in the last example ($0.781), then HBA1c testing would absolutely dominate FPG testing, as it would be less costly but more effective. In that scenario, the new HBA1c would be a more desirable option compared to FPG testing. However, if average transport costs were less than $0.07, the overall cost of FPG testing would fall below that of HBA1c testing. This would result in FPG testing absolutely dominating FPG testing.

## Discussion

In this study, we conducted a cost-effectiveness analysis comparing HBA1c to FPG POC testing. We found that despite the provider costs for HBA1c POC testing being greater than the provider costs of FPG POC testing by more than threefold, HBA1c testing dominated the FPG POC test from a societal perspective.

The cost-effectiveness of HBA1c testing in this study is attributable to its much lower patient costs than those of FPG testing, which were almost twice the patient costs of HBA1c testing. Patient costs contributed 65% to HBA1c testing compared to 90% for FPG testing. Adoption of HBA1c could mitigate out-of-pocket expenses, which could be a major barrier to diabetes screening. Conversely, improved access to diabetes screening could reduce transport and meal costs, mitigating the patient costs pertaining to FPG testing and making the latter a more desirable option.

We did not find any economic evaluation of the two tests that was conducted for an African population. However, we found one similar study comparing the cost effectiveness of HBA1c to that of FPG screening strategies, which was conducted in England [13]. In England, Gillett et al. [13] compared the cost effectiveness of HBA1c to that of FPG screening. However, the Gillett study was based on a provider perspective. This involved the cost of consumables, staff time, laboratory processing costs, and the costs of medication and other treatments. Additionally, health utility measures were applied to incident events to estimate lifetime discounted costs and quality-adjusted life years (QALYs). Their conclusions regarding HBA1c being the more cost-effective option mirrored our findings. This may be attributed not only to the patient spectrum in the respective study populations [27] but also to differences in time horizons and the choice of QALYs as the effectiveness measure.

In the Gillett study, glucose-related hazard ratios were calculated to model diabetes and cardiovascular complications. This approach allowed for the microsimulation of the predictive capacity of the tests for cardiovascular complications over the life course. The better predictive capacity for cardiovascular complications of the HBA1c test compared to the FPG test [28] may have mitigated the projected costs for future complications.

The finding that HBA1c testing is more cost-effective than FPG testing from a societal perspective is reassuring of efforts to improve early diabetes detection in this setting in the short term. This means that despite the upfront costs of the HBA1c test, investment in this modality as the basis for Uganda’s early diabetes detection programme could lead to cost savings. In addition, the greater convenience of the HBA1c test, which results in patients being tested at any time of day, irrespective of their fasting status, could improve the uptake of diabetes screening. This approach could facilitate the identification of more individuals with diabetes than the FPG test.

However, as access to care improves in the long term, lower provider and transport costs for a repeat visit would favour FPG testing. In addition, greater sensitization is likely to normalize regular wellness checks. With time, individuals are likely to know of the fasting pretest requirement for FPG testing, which would further reduce patient costs.

Assuming current costs for HBA1c testing, improvements in HBA1c sensitivity could result in testing using HBA1c being more effective as well as less costly. This state of absolute dominance of the HBA1c by the FPG would put HBA1c testing in the southeastern quadrant of the cost effectiveness plane, making it a more desirable option from an economics standpoint. In this case, HBA1c would be considered the most cost-effective option. Moreover, the higher sensitivity of the HBA1c test would result in a lower false negative fraction, improving diagnosis and potentially enrolment into care.

A strategy combining both the HBA1c and FPG tests could be implemented in such a way that HBA1c testing services are made available at secondary facilities while at the same time setting up FPG testing services at lower-level health facilities. Providing FPG services at lower-level facilities would mitigate patient costs, reducing the overall cost of FPG testing, even if patients had to make multiple screening visits. This strategy has the potential to reduce the ICER, making diabetes screening more affordable.

Our study has several limitations. The FPG sensitivity, specificity, and prevalence used to compute the proportion of patients correctly diagnosed were based on plasma glucose levels extrapolated from whole blood specimens using the Accu-Chek Active glucometers. The automatic calibration by glucometers to read plasma glucose from whole blood specimens is limited by the challenge of ensuring accuracy throughout the entire range of glycemia. Moreover, the calibration is proprietary and may not be transferrable to other glucometer brands [29]. This limits the generalizability of our findings to the Accu-Chek Active glucometer brand and not necessarily other glucometer brands. Additionally, although standard cut-offs are based on venous blood glucose, our measurements were based on capillary blood values. Capillary and venous blood glucose blood levels may differ due to factors such as nutritional status, physical activity, hydration status, stress levels and temperature, which were not accounted for in this study. Similarly, we did not adjust for potential variation in HBA1c levels due to racial and ethnic diversity. The vast genetic diversity inherent to East Africa would have involved the use of novel genomic techniques and a much larger sample size.

As noted above, the proportion of patients correctly diagnosed, which was the outcome measure for our analysis, is better suited to capturing test performance in the short term. The findings may not capture the full benefit of early diabetes diagnosis, as treatment outcomes and complications were excluded. Another limitation is the use of patient cost estimates from individuals already diagnosed with diabetes, as they are likely to be more homogenous than the general outpatient population that would be candidates for diabetes screening. However, it is unlikely that their expenses for food, lost productivity and transport would differ markedly from those of general outpatients.

Another limitation was that this study did not account for missed screening appointments in scenarios where FPG testing is used for diabetes screening. Missed screening appointments are likely to result in delayed diagnosis leading to poor clinical outcomes and higher provider and patient costs. Because this occurs in the future, it could not be accounted as the time horizon for the current study was a single testing cycle. Finally, we did not collect data on the pregnancy status of the women who participated in the study, making it impossible to rule out gestational diabetes.

This study has several strengths. First, the testing approach used mirrors an opportunistic screening strategy. Opportunistic screening within the healthcare system is more cost-effective than mass screening [30]. Opportunistic screening may lead to greater case yields and facilitate the follow-up of diagnosed patients compared with mass screening [31].

Second, we used FPG and HBA1c POC tests rather than central laboratory analysers. While POC testing typically incurs higher per-test costs than central laboratory testing in terms of reagents and operational expenses, the initial investment for POC devices is generally lower due to their smaller size and simpler infrastructure requirements. The WHO recommends POC testing in underserved settings where central laboratory facilities are unavailable. By providing a snapshot of the economic evidence for POC-related diabetes, our findings can inform efforts [4, 5] to develop POC-related diabetes diagnostic technologies for use in rural Uganda and similar low-income settings.

## Conclusion

The findings of this study that HBA1c testing was more cost effective than FPG testing from a societal perspective support efforts to improve early diabetes detection in this setting. This means that despite the upfront costs of the HBA1c test, investment in this modality as the basis for Uganda’s early diagnostic programme could lead to cost savings as well as facilitating the identification of more individuals with diabetes. In addition, the greater convenience of the HBA1c test, which results in patients being tested at any time of day, irrespective of their fasting status, could improve the use of diabetes testing and reduce the burden of undiagnosed diabetes.

## Supplementary Information

Supplementary Material 1.

Supplementary Material 2.

Supplementary Material 3.

Supplementary Material 4.

Supplementary Material 5.

## Data Availability

The datasets supporting the conclusions of this article are included within the article and its additional files).
