# Supplementary material for: Glycated haemoglobin versus fasting plasma glucose for type 2 diabetes point of care screening: a decision model cost-effectiveness analysis
Source: BMC Health Serv Res. 2025 May 9;25:664. doi: 10.1186/s12913-025-12840-4 (PMC12063251; doi:10.1186/s12913-025-12840-4)
Supplement: Supplementary file 1 — Supplementary Material 1. [file 12913_2025_12840_MOESM1_ESM.docx]

***Supplementary tables 1-6: Estimation of the costs of FPG testing***

Total no. of tests = 1,659

Exchange Rate: 1 USD = UGX 3790 (BOU, 2023)

Consumer Price Index (CPI) (UBOS, 2023)

June 2019: 107.32

August 2023: 127.51

***Supplementary table 1: Personnel costs during FPG testing***

| *Position and role* | *Number of personnel* | *Gross Salary (per person) (UGX)* | *Average time for conducting an FPG testing (minutes)* | *Personnel cost per test* (UGX)* | Total unadjusted personnel cost (UGX) | Total unadjusted personnel cost (USD) | Total personnel cost adjusted for inflation (USD) | **Unit cost adjusted for inflation (USD)** |
| --- | --- | --- | --- | --- | --- | --- | --- | --- |
| Nurse conducting FPG testing | 1 | 613,158 | 2.0 | 10.00 | 16,590 | 4.377 | $5.201 | **$0.003** |

*Assuming 260 working days per year and 8 hours per day.

***Supplementary table 2: Equipment costs during FPG testing***

| *Item* | *Cost per pc* | *Quantity* | *Total Cost* | *Current Price (USD)* | *Useful Life (yrs)* | *Annuity Factor** | *Annual Cost* | *Part Year^§^* | *Total Adjusted Cost* | *% for Intervention* | *Total annualized cost (US$)* | *Total cost adjusted for Intervention (US$)* | **Unit cost adjusted for inflation (USD)** |
| --- | --- | --- | --- | --- | --- | --- | --- | --- | --- | --- | --- | --- | --- |
| Accuchek active glucometers | UGX 80,000 | 1 | UGX 80,000 | $21.11 | 5 | 4.580 | $4.610 | 0.69 | $3.190 | 100% | $3.190 | $3.790 | **0.002** |

*The cost of the glucometer was annualized to account for the opportunity cost of paying for the glucometes upfront, yet the corresponding benefits are spread out over its useful life.

^§^Based on the duration of the screening intervention, i.e., 179 out of 260 working days.

***Supplementary table 3: Power costs during FPG testing***

| *Item* | *Total required* | *Unit cost* | *Total Cost, UGX* | *Total in USD* | *% for Intervention* | *Total unadjusted cost (US$)* | *Total adjusted cost (US$)* | **Unit cost adjusted for inflation (USD)** |
| --- | --- | --- | --- | --- | --- | --- | --- | --- |
| Batteries | 2 | 4,000.00 | 8,000 | $2.111 | 100% | $2.111 | $2.508 | **0.002** |

***Supplementary table 4: Test kits costs during FPG testing***

| *Item* | *Cost per packet* | *Total number of packets used* | *Total no. of test strips used* | *Total unadjusted cost (UGX)* | *Total cost, unadjusted (US$)* | *Total cost adjusted for inflation (US$)* | **Unit cost adjusted for inflation (USD)** |
| --- | --- | --- | --- | --- | --- | --- | --- |
| **Glucometer test strips (1x50 test strips)** | **67,420** | **46** | **2300** | **3,101,320** | **818.290** | **972.234** | **0.586** |

***Supplementary table 5: Costs of consumables during FPG testing***

| *Item* | *Units* | *Cost per packet* | *Total number of packets used* | *Full unadjusted cost (UGX)* | *Full unadjusted cost (US$)* | *Full cost adjusted for inflation (USD)* | ***Unit cost adjusted for inflation (USD)*** |
| --- | --- | --- | --- | --- | --- | --- | --- |
| Disposable gloves, box | Pair of gloves | 12,657 | 23.00 | 291,111 | 76.810 | 91.260 | 0.055 |
| 70% Alcohol swabs | 1 pc | 6,626.00 | 21.00 | 139,146 | 36.714 | 43.621 | 0.026 |
| Accu-Chek Safe-T-pro Uno lancets | 1 pc | 55,000.00 | 11.00 | 605,000 | 149.631 | 177.781 | 0.107 |
| **Sub-total, Cost of consumables** | |  |  |  |  | **312.662** | **0.188** |

***Supplementary table 6: Estimation of patient costs*** (Estimated from Shiri *et al.* (2021))

Consumer Price Index (CPI) (UBOS, 2023)

June 2020: 110.95

August 2023: 127.51

| **Item** | **Expense (2019 USD)** | **Expense (2023 USD)** |
| --- | --- | --- |
| Transport^⁑^ | 2.32 | 2.666 |
| Lost productivity* | 1.35 | 1.551 |
| Meals | 2.775 | 3.189 |
| **Total patient costs for PFG testing** |  | **7.407** |

^⁑^ Inflated by 100% to account for the fact that practically all patients due for FPG testing were not being in a fasted state when the first interfaced with study staff. They all had to return on a second day to provide a fasting capillary blood sample for FPG testing.

*Inflated by 50% to account for the tendency for patients undergoing FPG testing to spend less time at the facility on the second visit compared to the first visit.

**REFERENCES**

BOU. (2023). *Bank of Uganda| Home*. https://www.bou.or.ug/bou/bouwebsite/BOU-HOME

Shiri, T., Birungi, J., Garrib, A. V., Kivuyo, S. L., Namakoola, I., Mghamba, J., Musinguzi, J., Kimaro, G., Mutungi, G., Nyirenda, M. J., Okebe, J., Ramaiya, K., Bachmann, M., Sewankambo, N. K., Mfinanga, S., Jaffar, S., & Niessen, L. W. (2021). Patient and health provider costs of integrated HIV, diabetes and hypertension ambulatory health services in low-income settings—An empirical socio-economic cohort study in Tanzania and Uganda. *BMC Medicine*, *19*(1), 230. https://doi.org/10.1186/s12916-021-02094-2

UBOS. (2023). *CPI Excel Tables for CPI August 2023* [dataset]. https://www.ubos.org/wp-content/uploads/statistics/CPI_Excel_Tables_for_CPI_August_2023.xlsx
