# Supplementary material for: Glycated haemoglobin versus fasting plasma glucose for type 2 diabetes point of care screening: a decision model cost-effectiveness analysis
Source: BMC Health Serv Res. 2025 May 9;25:664. doi: 10.1186/s12913-025-12840-4 (PMC12063251; doi:10.1186/s12913-025-12840-4)
Supplement: Supplementary file 2 — Supplementary Material 2. [file 12913_2025_12840_MOESM2_ESM.docx]

***Supplementary tables 7-11: Estimation of the costs of HBA1c testing***

Total no. of tests = 1,659

Exchange Rate: 1 USD = UGX 3790 (BOU, 2023)

Consumer Price Index (CPI) (UBOS, 2023)

August 2023: 127.51

June 2020: 110.95

June 2019: 107.32

***Supplementary table 7: Personnel costs for HBA1c testing***

| *Position and role* | *Number of personnel* | *Gross Salary per person*  *(UGX)* | *Average time for conducting an FPG testing (minutes)* | *Personnel cost per test* (UGX)* | Total unadjusted personnel cost (UGX) | Total unadjusted personnel cost (USD) | Total personnel cost adjusted for inflation (USD) | **Unit cost adjusted for inflation (USD)** |
| --- | --- | --- | --- | --- | --- | --- | --- | --- |
| Laboratory technician conducting HBA1c testing | 1 | 613,158 | 6.0 | 29.480 | 48,907.320 | 12.904 | 15.332 | **0.009** |

*Assuming 260 working days per year and 8 hours per day.

***Supplementary table 8: Equipment costs for HBA1c testing***

| *Item* | *Cost per pc* | *Quantity* | *Total Cost* | *Current Price (USD)* | *Useful Life (yrs)* | *Annuity Factor* | *Annual Cost (USD)* | *Part Year?* | *% for Intervention* | *Total annualized Cost (USD)* | Total annualized cost adjusted for inflation (USD) | **Unit cost adjusted for inflation (USD)** |
| --- | --- | --- | --- | --- | --- | --- | --- | --- | --- | --- | --- | --- |
| Cobas b101 HBA1c analyzer | 7,923,000.00 | 1 | 7,923,000.00 | 2,090.73 | 5 | 4.580 | 456.52 | 0.30 | 100% | 136.20 | **161.832** | **0.097** |
| 1 KVA Uninterruptible Power Supply (UPS) units | 1,127,811.00 | 1 | 1,127,811.00 | 297.61 | 5 | 4.580 | 64.98 | 0.30 | 100% | 19.39 | **23.038** | **0.014** |
| **Sub-total, HBA1c testing equipment** |  |  |  |  |  |  |  |  |  |  | **184.87** | **0.112** |

*The HBA1c equipment costs were annualized to account for the opportunity cost of paying for the equipment upfront, yet the corresponding benefits are spread out over its useful life.

*^‡^*The duration of HBA1c screening was adjusted relative to FPG testing. This was done partly to account for the longer daily service availability. Based on the study experience and our interviews with MoH staff, HBA1c testing services would be available for 6 hours, from 9.00am to 4.00pm, excluding the time for a 1-hour lunch break. This contrasts with FPG testing which is usually available for two hours from 9:00 am to 11:00 am.

The adjustment of HBA1c screening duration also served the purpose of accounting for the likely greater uptake of HBA1c screening due to its requirement for only a single patient visit. FPG testing, on the other hand, requires the patient to return in future for testing while in a fasting state. Because of this, some patients are likely not to return to the health facility for the FPG testing visit, increasing the duration required to test the same number of patients using FPG testing. Since testing was conducted on the next visit during the study, FPG testing was the main determinant of the overall screening duration. We hypothesize that the duration would be shorter if the screening were based on HBA1c testing alone. We assumed that in the context of routine care, about 30% of the patients would not return for FPG testing. For this reason, the total duration required to test the same number of patients using the HBA1c test was further reduced by 30%.

***Supplementary table 9: Costs of power during HBA1c testing***

The power consumption of the HBA1c POC system is 12V and 5A, according to the product specification.

| *Item* | *Voltage (V)* | *Current (A)* | *Power (W)* | *Time per test (min)* | *Energy consumption per test (kWh)* | *Cost per kWh (UGX)* | *Total unadjusted cost (UGX)* | Total unadjusted cost (USD) | **Total cost adjusted for inflation (USD)** | **Unit cost adjusted for inflation (USD)** |
| --- | --- | --- | --- | --- | --- | --- | --- | --- | --- | --- |
| Electric power | 12V | 5 | 60 | 6 | 0.006 | 250 | 2488.500 | 0.657 | **0.780** | **0.000** |

*Table 2D: Test kits*

| *Item* | *Cost per test, UGX* | *Number of test kits used* | *Total unadjusted cost (UGX)* | *Total unadjusted cost (USD)* | **Total cost adjusted for inflation (USD)** | **Unit cost adjusted for inflation (USD)** |
| --- | --- | --- | --- | --- | --- | --- |
| Cobas b101 test kits (1x10) | 6,762 | 1659 | 11,218,158 | 2,959.936 | 3,516.786 | **2.120** |

***Supplementary table 10: Costs of consumables during HBA1c testing***

| *Item* | *Units* | *Cost per packet* | *Total number of packets used* | *Full unadjusted cost (UGX)* | *Full unadjusted cost (US$)* | *Full cost adjusted for inflation (USD)* | ***Unit cost adjusted for inflation (USD)*** |
| --- | --- | --- | --- | --- | --- | --- | --- |
| Disposable gloves, box | Pair of gloves | 12,657 | 23.00 | 291,111 | 76.810 | 91.260 | 0.055 |
| 70% Alcohol swabs | 1 pc | 6,626.00 | 21.00 | 139,146 | 36.714 | 43.621 | 0.026 |
| Accu-Chek Safe-T-pro Uno lancets | 1 pc | 55,000.00 | 11.00 | 605,000 | 149.631 | 177.781 | 0.107 |
| **Sub-total, Consumables** |  |  |  |  |  | **312.662** | **0.188** |

***Supplementary table 11: Patient costs*** ***during HBA1c testing*** *(estimated from Shiri et al.* (2021)*)*

Consumer Price Index (CPI) (UBOS, 2023)

August 2023: 127.51

June 2020: 110.95

| **Item** | **Expense (2019 USD)** | **Expense (2023 USD)** |
| --- | --- | --- |
| Transport | 1.160 | 1.333 |
| Lost productivity | 0.900 | 1.034 |
| Meals | 1.850 | 2.126 |
|  |  | **4.493** |
