# Supplementary material for: Glycated haemoglobin versus fasting plasma glucose for type 2 diabetes point of care screening: a decision model cost-effectiveness analysis
Source: BMC Health Serv Res. 2025 May 9;25:664. doi: 10.1186/s12913-025-12840-4 (PMC12063251; doi:10.1186/s12913-025-12840-4)
Supplement: Supplementary file 3 — Supplementary Material 3. [file 12913_2025_12840_MOESM3_ESM.docx]

**Tornado diagrams for the ICER and Incremental Effectiveness of HBA1c vs FPG testing**


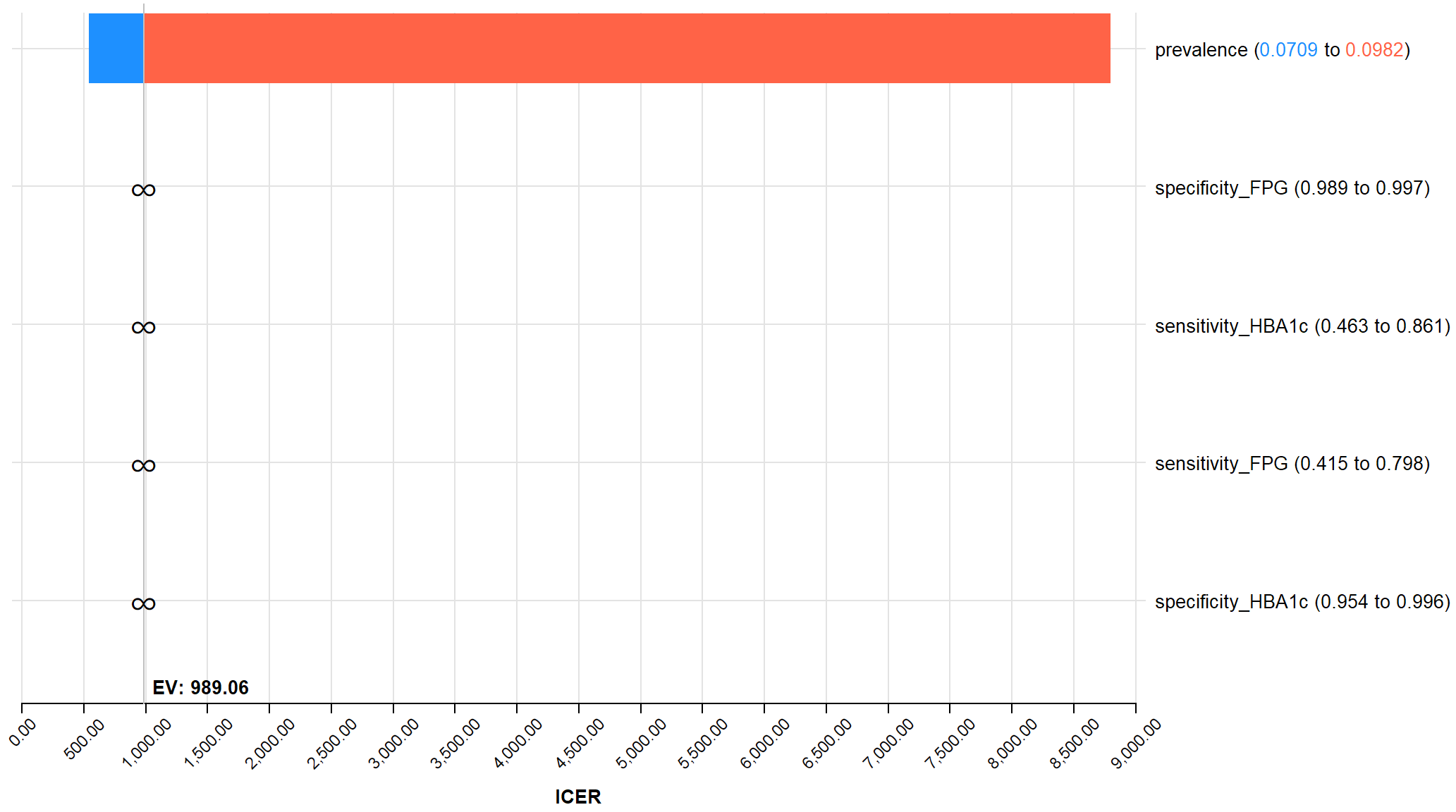


*Supplementary Fig 1: Tornado Diagram: ICER, HBA1c testing vs. FPG testing*

*
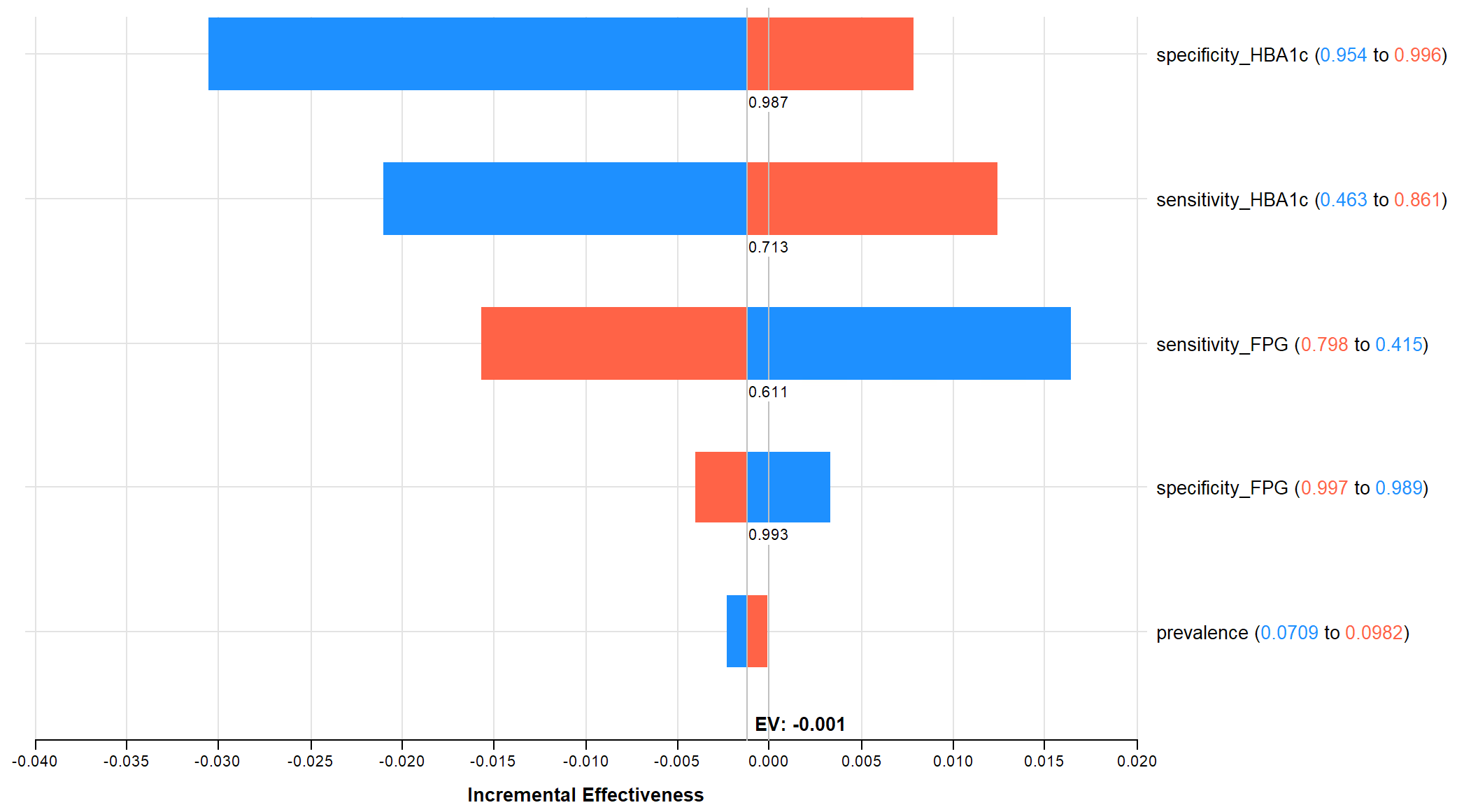
*

*Supplementary Fig 2: Tornado diagram for the Incremental Effectiveness of HBA1c testing vs FPG testing*
