# Supplementary material for: Glycated haemoglobin versus fasting plasma glucose for type 2 diabetes point of care screening: a decision model cost-effectiveness analysis
Source: BMC Health Serv Res. 2025 May 9;25:664. doi: 10.1186/s12913-025-12840-4 (PMC12063251; doi:10.1186/s12913-025-12840-4)
Supplement: Supplementary file 5 — Supplementary Material 5. [file 12913_2025_12840_MOESM5_ESM.docx]

**Questionnaire to inform the costing of FPG, HBA1c testing and patient costs**

| **#** | **Research Assistant** | **Response** |
| --- | --- | --- |
| 1. | Based on your experience, what proportion of patients were in fasting state, enabling you to collect a fasting capillary blood sample for FPG testing on day 1? | └──┘ |
| 2. | Based on your experience, what proportion of patients were in a fasting state, enabling you to collect a fasting capillary blood saple for FPG testing on day 2? | └──┘ |
|  | Based on your experience, how much time did patients spend at the health facility during their testing visit compared to their initial pre-test visit? | └──┘ |
|  | **In-charge of the outpatient department** |  |
| 4. | What is the cadre of staff that would do the HBA1c test if it were offered routinely as part of an outpatient opportunistic diabetes screening package at the hospital? | └──┘ |
| 5. | What is the cadre of staff that would do the FPG test if it were offered routinely as part of an outpatient opportunistic diabetes screening package at the hospital? | └──┘ |
| 6. | How many of those staff would be assigned to conduct HBA1c testing, if the test were offered routinely as part of an outpatient opportunistic diabetes screening package at the hospital? | └──┘ |
| 7. | How many of those staff would be assigned to conduct HBA1c testing, if the test were offered routinely as part of an outpatient opportunistic diabetes screening package at the hospital? | └──┘ |
| 8. | Based on current working procedures, at what time of the day would testing start? | └──┘ |
| 9. | Based on current working procedures, at what time of the day would testing cease? | └──┘ |
